# Magnetic Resonance Imaging of Postoperative Fracture Healing Process without Metal Artifact: A Preliminary Report of a Novel Animal Model

**DOI:** 10.1155/2016/1429892

**Published:** 2016-03-31

**Authors:** Zhe Jin, Yuheng Guan, Guibo Yu, Yu Sun

**Affiliations:** ^1^Department of Orthopaedics, First Hospital of China Medical University, 155 Nan Jing Bei Street, Heping District, Shenyang 110001, China; ^2^Department of Radiological Diagnosis and Interventional Treatment, First Hospital of China Medical University, Shenyang 110001, China

## Abstract

*Background*. Early radiological diagnosis and continual monitoring are of ultimate importance for timely treatment of delayed union, nonunion, and infection after bone fracture surgery. Although magnetic resonance imaging (MRI) could provide superior detailed images compared with X-ray and computed tomography (CT) without ionizing radiation, metal implants used for fracture fixation lead to abundant artifacts on MRI and thus prohibit accurate interpretation. The authors develop a novel intramedullary fixation model of rat femoral fracture using polyetheretherketone (PEEK) threaded rods and investigate its feasibility for in vivo MRI monitoring of the fracture healing process without artifact.* Methods*. Femoral fractures of 3 adult male Sprague-Dawley rats were fixed with intramedullary PEEK threaded rods. X-ray and MRI examinations were performed at day 7 postoperatively. Radiological images were analyzed for the existence of artifact interruption and postoperative changes in bone and peripheral soft tissue.* Results*. Postoperative plain film revealed no loss of reduction. MRI images illustrated the whole length of femur and peripheral tissue without artifact interruption, and the cortical bone, implanted PEEK rod, and soft tissue were clearly illustrated.* Conclusion*. This preliminary study introduced a novel rat model for in vivo MRI monitoring of the fracture healing process without metal artifact, by using intramedullary fixation of femur with PEEK threaded rod.

## 1. Introduction

Trauma and osteoporosis lead to millions of bone fractures each year around the world, and with an aging population the incidence rate will continue increasing in the foreseeable future [1]. Although great advances have been achieved in surgical treatment, postoperative delayed union, nonunion, and infections are still grand challenges faced by orthopaedic surgeons [2]. To prevent long-term adverse effects caused by these complications, early radiological diagnosis and continual monitoring are of ultimate importance to guarantee timely intervention.

For decades, the formation of bone callus and the visibility of fracture line on X-ray or computed tomography (CT) images have been viewed as the most important criteria for clinical evaluation, but these techniques are only suitable for examining mineralized cortical bone, which does not allow early detection during the first several weeks after surgery [3]. Moreover, harmful effects associated with ionizing radiation caused by plain film and CT also limit their application for continual monitoring of fracture sites. As to major tissue components for early healing process, such as newly formed unmineralized bone, bone marrow in medullary cavity, and peripheral soft tissue with less X-ray contrast properties, magnetic resonance imaging (MRI) could provide superior detailed images from the beginning to bone union with excellent resolution and thin slices in any direction but without radiation injury, being recognized as the most appropriate diagnostic method for depicting the dynamic repairing, inflammatory, and infectious process [4]. Besides, high resolution MRI could also provide detailed information on bone neurovascularization and fibrovascular tissue ingrowth adjacent to medical implants comparable to anatomical dissection and histological studies [5, 6]. However, metal implants used for fracture fixation lead to abundant artifacts on MRI and thus prohibiting accurate interpretation of postoperative images, and MRI without artifact is needed for further research.

Fortunately in recent years, with leaping advances in biomaterials science, new polymeric materials have been developed and come into clinical applications with excellent mechanical properties and biosafety and without artifact interruption on MRI. We have previously developed a rat model of tibia fracture fixation with polyetheretherketone (PEEK) threaded rod, which allowed early postoperative full-weight bearing activities that resemble clinical treatment of human long bone fractures without using surgical metal implants [7]. This preliminary study aimed to develop a novel intramedullary fixation model of rat femoral fracture using PEEK threaded rod and to investigate its feasibility for in vivo MRI monitoring of the fracture healing process without artifact, which may help lab researchers and clinicians in future diagnostic and therapeutic research of postoperative operative complications of orthopaedic surgeries.

## 2. Materials and Methods

### 2.1. PEEK Threaded Rod Preparation

PEEK threaded rods were prepared as described in previous publication [7]. The rods were 2 mm in diameter and 100 mm in length with standard pitch (0.4 mm). All PEEK rods were rinsed ultrasonically with 70% ethanol for 24 hours and then underwent ethylene oxide sterilization according to operation standards used for surgical instruments. Animal experiment protocols were first approved by the ethical committee in the authors' institution.

### 2.2. Animals

Three skeletally mature male Sprague-Dawley rats (280–300 g) provided by the Laboratory Animal Center of China Medical University were kept in plastic cage with free access to food and water, at constant room temperature with a 12-hour day/night cycle for 7 days before operation as accommodation.

### 2.3. Surgical Procedure

**Figure 1:**
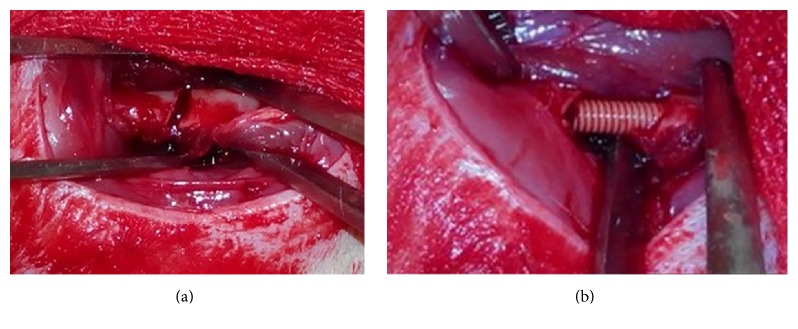
(a) Intraoperative image showing the site of osteotomy. (b) Intraoperative image showing the insertion of PEEK threaded rod for intramedullary fixation.

Before surgery, animals received intraperitoneal injections of pentobarbital sodium (Merck, Darmstadt, Germany) for general anesthesia (50 mg/kg). The left hind leg was shaved with electric scissor and disinfected with povidone. First, a 1 cm anteromedial knee incision was made and the patella was released laterally to expose the distal femoral condyle, and a 2 mm diameter intercondylar bone tunnel was drilled with a depth of 20 mm. Then, another 1.5 cm longitudinal lateral skin incision was made between anterior and posterior muscle groups to expose the left femur, and the level of osteotomy was set in the middle of femur shaft (Figure 1(a)). After transverse osteotomy, a 20 mm PEEK threaded rod (Figure 1(b)) was inserted by a press-fit maneuver from distal to proximal, and care was taken to ensure rigid fixation of bone osteotomy without extra bone damage, followed by wound irrigation with normal saline. And the muscle fascia and skin were sutured interruptedly.

### 2.4. Postoperative Imaging

After 1 week, all animals received general anesthesia as stated above and postoperative plain film and MRI were obtained. A standard imaging module of an X-ray unit (Arcadis; Siemens, Germany) was used for X-ray examination, and in vivo MRI was imaged at 3.0T MRI scanner (Signa HDxt, GE Healthcare, USA) using a rat imaging coil with sagittal projections and FSE *T*2-weighted (TR = 4000, TE = 100) sequences with a 40 × 40 mm^2^ field of view, 2 mm slice thickness, 0.2 mm slice spacing, and 256 × 256 matrix. The total scan time was approximately 340 seconds. Regions of interest were analyzed on MRI including the cortical bone and medullary cavity around osteotomy site and signal intensity changes of surrounding soft tissue. All animals were euthanized via overdose injection of pentobarbital after MRI experiment.

## 3. Results

**Figure 2:**
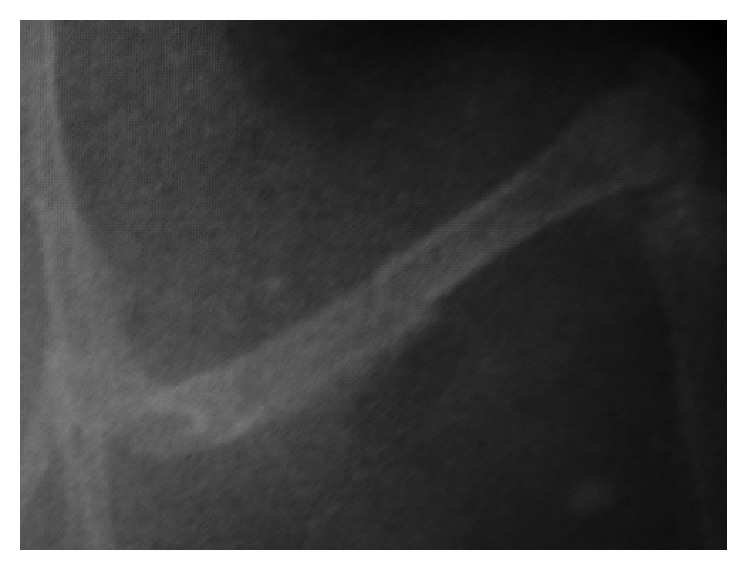
Postoperative X-ray examination illustrating no loss of reduction between fracture sites and the level of osteotomy in the middle of femur shaft.

**Figure 3:**
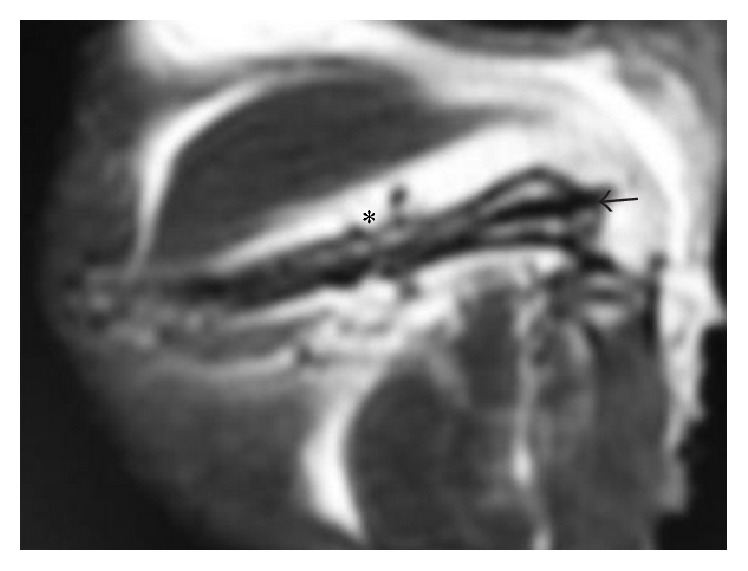
Postoperative MRI illustrating the femur, PEEK rod, and peripheral soft tissue without artifact (arrow: PEEK threaded rod; asterisk: the site of osteotomy).

There were no postoperative wound complications observed during the study. Postoperative plain films revealed no loss of reduction or malalignment between fracture sites, and the site of osteotomy was well defined (Figure 2). The postoperative MRI images illustrated the whole length of left femur without artifact interruption. The cortical bone and implanted PEEK rod (arrow in Figure 3) in the medullary cavity were of low signal on MRI due to lack of free protons for resonance, and a region of discontinuity of the low signal representing cortical bone damage could be identified at the level of osteotomy (asterisk in Figure 3), and increased signal representing adjacent soft tissue edema and inflammation was clearly illustrated.

## 4. Discussion

Although X-ray and CT have been recognized as the most important radiological tools for postoperative diagnosis of orthopaedic surgical complications, increasing evidence has suggested that MRI could demonstrate higher sensitivity for detecting osseous changes, ectopic bone formation, myelopathies, soft tissue inflammation, and neurovascular changes, allowing assessment of morphological, physical, and chemical properties such as implant position, tissue hydration, and water/lipid ratios [4, 8, 9]. High resolution MRI device used mostly in lab studies may acquire image slices at a thickness of less than 100 *μ*m and cellular level analysis correlated with traditional histological studies, enabling researchers to continuously visualize the cell and tissue behavior around the implant in vivo for evaluation of tissue-engineering products [3]. Besides, MRI is a noninvasive and ideal technique without X-ray radiation damage to patients. For instance, Park et al. and De Potter et al. used MRI examination to evaluate the extent of fibrovascular ingrowth into implanted polyethylene or hydroxyapatite ocular prosthesis and early signs of immune rejection [6, 10]. However, in the area of orthopaedic research no further progress has been made because of the existence of metallic artifact. Although many orthopaedic metal materials such as titanium are not ferromagnetic (MR safe), this paramagnetic hardware is still of large magnetic susceptibility differences with the surrounding tissue, so both ferromagnetic and paramagnetic metal implants can induce inhomogeneity in the static magnetic field (*B*0). Moreover, the switching of gradient pulses in an MRI scanner could produce eddy currents in the metal implant, which is another cause of *B*0 inhomogeneity. The *B*0 inhomogeneity will result in image distortion as metal-related artifacts, severely reducing the quality of examinations and obscuring anatomical regions which may lead to false diagnosis or evaluation [11, 12].

PEEK as a polymeric material is nonmagnetic and radiofrequency transparent and does not generate MRI artifact. It is chemically stable and nontoxic for both animal research and clinical treatment [13], also with excellent mechanical strength, ideal elastic modulus similar to cortical bone, and radiological translucency [14]. There are other studies published selecting PEEK plates for fracture fixation, but metal screws were still used in combination with PEEK [15]. To solve the problem, we have previously machined thread on PEEK rods to provide press-fit compression for intramedullary bone fixation and developed a tibia fracture healing model with simple operation procedure, good tissue protection, and excellent X-ray translucency [7]. Our threaded rods design on PEEK materials may also provide simple but effective fixation for clinical treatment of certain human tubular bones, especially for non-weight-bearing regions in upper extremities such as clavicle, radius, ulna, and metacarpal bones. In this study, we further investigated its application for rat femur fixation and in vivo postoperative MRI examination. Rat femurs were selected in this study to provide better images of the whole bone as illustrated in Figure 3, considering that the anatomical curvature of rat tibia may add unnecessary technical difficulties. To our knowledge, this is the first report of noninvasive in vivo MRI monitoring of long bone fracture healing using pure PEEK polymeric material for fixation.

This preliminary study has some limitations. We obtained MRI images using *T*2-weighted sequences without MR contrast one week postoperatively. Future experiments could include more MRI sequences and contrast agents at more time points, which will involve more animals in study and allow visualization of dynamic cellular and tissue behavior during fracture healing following surgical implantation. Moreover, other animal models with impaired fracture healing process could be evaluated in vivo using our fixation method, such as type-2 diabetes, osteoporosis, critical-size bone defect, tumor, and other inflammatory or infectious diseases.

## 5. Conclusions

This preliminary study introduced a novel rat model for in vivo MRI monitoring of the fracture healing process without metal artifact, through intramedullary fixation of femur using PEEK threaded rod. This animal model may provide a new diagnostic tool in future studies on postoperative complications of orthopaedic surgeries.
